# Supplementary material for: High-fidelity spin and optical control of single silicon-vacancy centres in silicon carbide
Source: Nat Commun. 2019 Apr 26;10:1954. doi: 10.1038/s41467-019-09873-9 (PMC6486615; doi:10.1038/s41467-019-09873-9)
Supplement: Supplementary file 1 — Supplementary Information [file 41467_2019_9873_MOESM1_ESM.pdf]

Supplementary Information:

High-fidelity spin and optical control of single silicon vacancy centres in silicon carbide

Roland Nagy et al.

Supplementary Note 1. Identification of single silicon vacancy centres.

To confirm that measurements were performed on single silicon vacancy defects, we performed Hanbury Brown and Twiss (HPT) experiments[1]. To this end, the defect was excited using the off-resonant laser (730 nm) and we recorded the second-order correlation function $g^{(2)}(\tau)$ of the fluorescence emission. A typical result is shown in Supplementary Figure 1 in which $g^{(2)}(\tau = 0) \ll 0.5$. This stands as a clear proof that a single photon emitter is investigated[2].

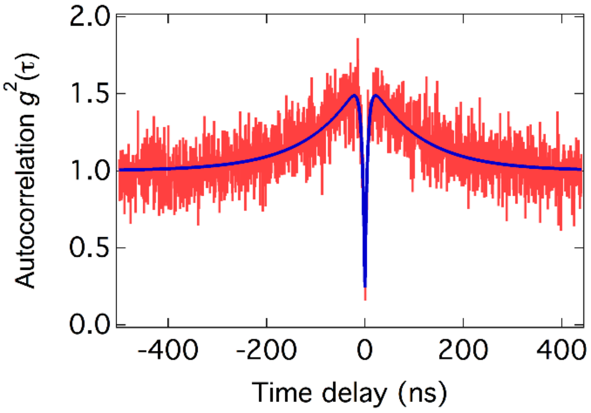

Supplementary Figure 1: Second-order autocorrelation function. Data are represented in red, and the blue line is a fit assuming a three-level model function: $g^{(2)}(\tau) = \left(1 - \beta \cdot e^{-\frac{|\tau|}{\tau_1}} - (1-\beta) \cdot e^{-\frac{|\tau|}{\tau_2}}\right) \cdot 1/N + (N-1)/N$, in which $g^{(2)}(\tau = 0) = (N-1)/N$, and N is the number of single photon emitting emitters. From this fit, we extract the following parameters: $g^{(2)}(\tau = 0) = 0.24 \pm 0.06$, $\tau_1 = 5.5 \pm 0.4\, ns$, and $\tau_2 = 103.7 \pm 3.9\, ns$.

Supplementary Note 2. Inferring the sign of the excited state zero field splitting.

Using a wavemeter (Coherent WaveMaster) with a resolution of 0.1 GHz, we inferred that the A_2 optical transition is the one at higher energy. We now want to infer whether this transition links the spin sublevels $|\pm\frac{3}{2}\rangle_{\mathrm{gs}}$ (resulting in a positive sign of D_{es}) or the sublevels $|\pm\frac{1}{2}\rangle_{\mathrm{gs}}$ (resulting in a negative sign of D_{es}). For this, the experimental results of the Rabi oscillations between $|-\frac{1}{2}\rangle_{\mathrm{gs}}$ and $|+\frac{1}{2}\rangle_{\mathrm{gs}}$ are relevant (see Figure 3(b) in the main text).

We now consider both cases of D_{es} (positive and negative):

Assuming that D_{es} is positive, the A_2 optical transition links the $m_{\mathrm{S}} = \pm\frac{3}{2}$ sublevels,

such that the ground state spin initialization procedure (laser excitation on the A_2 transition and continuous MW_3 excitation in order to depopulate $|+\frac{1}{2}\rangle_{\mathrm{gs}}$) leads to near deterministic population of $|-\frac{1}{2}\rangle_{\mathrm{gs}}$. Thereafter, MW_2 is applied for τ_{Rabi}, followed by a population swap (π-pulse at MW_3) between $|+\frac{1}{2}\rangle_{\mathrm{gs}}$ and $|+\frac{3}{2}\rangle_{\mathrm{gs}}$, and eventual population readout of the states $|\pm\frac{3}{2}\rangle_{\mathrm{gs}}$. This experiment should lead to clearly visible Rabi oscillations, starting at a minimum intensity.

Assuming on the other hand that D_{es} is negative, then A_2 links the $m_{\mathrm{S}} = \pm\frac{1}{2}$ sublevels, such that the ground state spin initialization procedure would lead to a near deterministic population of $|-\frac{3}{2}\rangle_{\mathrm{gs}}$. Thereafter, MW_2 is applied for τ_{Rabi}, which does not alter the ground state spin population at all. In addition, the population swap π-pulse at MW_3 has also no effect. As a consequence, the eventual population readout of the states $|\pm\frac{1}{2}\rangle_{\mathrm{gs}}$ should lead to no observable signal.

The experimental results in Figure 3(b) in the main text clearly support therefore that the excited state zero field splitting is positive, *i.e.* $2\cdot D_{\mathrm{es}} = 985 \pm 10\ \mathrm{MHz}$.

We note that our *ab initio* calculations[5] resulted in positive zero-field constant both for the ground and excited states that strongly supports the analysis of experimental results.

Supplementary Note 3. Landé g-factor and optical transition energies of the V1 excited state.

The spin Hamiltonian of ground and excited states with an external axial magnetic field is:

$$H_{\mathrm{gs,es}} = D_{\mathrm{gs,es}} \cdot {S_z}^2 + g_{\mathrm{gs,es}}\, \mu_{\mathrm{B}}\, B_0 \cdot S_z. \qquad (1)$$

Here, the subscripts gs and es denote ground and excited states, respectively. $2 \cdot D_{\mathrm{gs,es}}$ denotes the zero field splitting, S_z is the spin projection operator in the *z*-direction, $g_{\mathrm{gs,es}}$ is the Landé g-factor, μ_{B} is the Bohr magneton, and B_0 the strength of the axial magnetic field.

Diagonalization of the Hamiltonian leads to four spin-conserving optical transition energies:

$2 \cdot (D_{\mathrm{es}} - D_{\mathrm{gs}}) + \frac{3}{2} \cdot \mu_{\mathrm{B}} \cdot B_0 \cdot (g_{\mathrm{es}} - g_{\mathrm{gs}}) + \Delta E_{\mathrm{gs,es}}$ for transitions between $m_{\mathrm{S}} = +\frac{3}{2}$ sublevels.

$\frac{1}{2} \cdot \mu_{\mathrm{B}} \cdot B_0 \cdot (g_{\mathrm{es}} - g_{\mathrm{gs}}) + \Delta E_{\mathrm{gs,es}}$ for transitions between $m_{\mathrm{S}} = +\frac{1}{2}$ sublevels.

$-\frac{1}{2} \cdot \mu_{\mathrm{B}} \cdot B_0 \cdot (g_{\mathrm{es}} - g_{\mathrm{gs}}) + \Delta E_{\mathrm{gs,es}}$ for transitions between $m_{\mathrm{S}} = -\frac{1}{2}$ sublevels.

$2 \cdot (D_{\mathrm{es}} - D_{\mathrm{gs}}) - \frac{3}{2} \cdot \mu_{\mathrm{B}} \cdot B_0 \cdot (g_{\mathrm{es}} - g_{\mathrm{gs}}) + \Delta E_{\mathrm{gs,es}}$ for transitions between $m_{\mathrm{S}} = -\frac{3}{2}$ sublevels.

Here, $\Delta E_{\mathrm{gs,es}} \approx 1.44\ \mathrm{eV}$ is the energy difference between ground and excited states. At zero magnetic field, one expects two pairwise degenerate spin-conserving optical transitions for the sublevels $m_{\mathrm{S}} = \pm\frac{1}{2}$ and $m_{\mathrm{S}} = \pm\frac{3}{2}$. For the latter transition (A_2 in our notation), we observe a linewidth (full width at half maximum (FWHM)) of $\Delta f_{B_0 = 0\ \mathrm{G}} = 87.6 \pm 1.6\ \mathrm{MHz}$. At $B_0 \neq 0\ \mathrm{G}$, one would expect to see four optical spin-conserving

transitions, provided that there is a sizeable difference in the Landé factors g_{gs} and g_{es}. As shown in Figure 1(c) in the main text, we do not observe any additional transitions at $B_0 = 92\ \mathrm{G}$. The linewidth of the A_2 transition remains essentially unchanged, *i.e.* $\Delta f_{B_0=92\ \mathrm{G}} = 87.7 \pm 1.6\ \mathrm{MHz}$. Consequently, we conclude that the change in linewidths is $\Delta f = \Delta f_{B_0=92\ \mathrm{G}} - \Delta f_{B_0=0\ \mathrm{G}} = 0.1 \pm 2.3\ \mathrm{MHz}$. We assume now that the optical transitions between the sublevels $m_S = \pm\frac{3}{2}$ show a Lorentzian profile, *i.e.* $I_\pm \propto \frac{\left(\frac{a}{2}\right)^2}{\left(f \pm \frac{f_0}{2}\right)^2 + \left(\frac{a}{2}\right)^2}$, in which $I_\pm$ denotes the intensity of the $m_S = \pm\frac{3}{2}$ transition, $a = \Delta f_{B_0=0\ \mathrm{G}}$ is the FWHM of each transition at $B_0 = 0\ \mathrm{G}$, f is the laser frequency offset with respect to the centre of the resonance, and $\pm\frac{f_0}{2}$ is the displacement of the $m_S = \pm\frac{3}{2}$ transition at $B_0 \neq 0\ \mathrm{G}$. There exists an analytical solution for the apparent FWHM of the sum of two displaced Lorentzian functions:

$$\Delta f_{\mathrm{Double\ Lorentz}} = \sqrt{2 \cdot f_0^2 - \sqrt{5 \cdot f_0^4 + 8 \cdot f_0^2 \cdot a^2 + 16 \cdot a^4}}. \qquad (2)$$

Using this equation, we infer a displacement of $f_0 = 0.2 \pm 11.6\ \mathrm{MHz}$. By using this result, we now infer the difference in ground and excited state Landé g-factors:

$$g_{\mathrm{es}} - g_{\mathrm{gs}} = \frac{\Delta f_{\mathrm{Double\ Lorentz}}}{3 \cdot \mu_{\mathrm{B}} \cdot B_0} = (0.5 \pm 30.0) \cdot 10^{-3}. \qquad (3)$$

Since previous studies[3] have already reported $g_{\mathrm{gs}} = 2.0028$, we determine the excited state Landé factor to be $g_{\mathrm{es}} = 2.0033 \pm 0.0300$.

In addition, as the ground and excited state *g*-factors have been determined to be nearly identical, if spin-flipping optical transitions $(|\Delta m_S| = 1)$ were allowed, they should appear at $\pm g \mu_{\mathrm{B}} B_0 \approx \pm 258\ \mathrm{MHz}$ compared to the spin-conserving transitions. However, such transitions have not been observed as shown in Figure 1(c).

Supplementary Note 4. Inhomogeneous broadening of optical resonance

In the main text, we report 60 MHz linewidth of single silicon vacancy resonant optical transition. Although this is close to the lifetime-limit, we still observe a small residual inhomogeneous broadening. We attribute the broadening to local electric field fluctuations originating from free carriers injected by ionized defects in proximity. Although the change in dipole moments by optical pumping is minimal, since it still can be coupled to the fluctuating electric fields, residual broadening takes place. As discussed in the Methods of the main text and S5&6 of the Supplementary Information, various defects exist in the tested samples which are created during the sample growth and post-process or by electron irradiation, such as shallow nitrogen impurities, and carbon vacancies. We prepared another sample, which was intentionally irradiated with a 10 times higher electron dose. This results in an overall higher concentration of defects, especially carbon vacancies. We show in Supplementary Figure 2 several absorption spectra of single V_{Si} defects in the high-dose irradiated sample (irradiation dose $10^{13}\ \mathrm{cm}^{-2}$). Optical excitation intensity levels were set to be under 1 W/cm^2 to avoid power broadening. We find an average linewidth of 160 MHz which is nearly three times larger than 60 MHz in the low-dose irradiated sample. Since the only different parameter in the low- and high-dose irradiated samples is the irradiation dose which differs by one order of magnitude, we attribute this broadening to the increased density of free carriers originating from dense defect clusters surrounding the tested silicon vacancies in the high-dose irradiated sample. This result shows that the high purity silicon carbide crystal is essential for achieving lifetime-limited linewidth of optical transition.

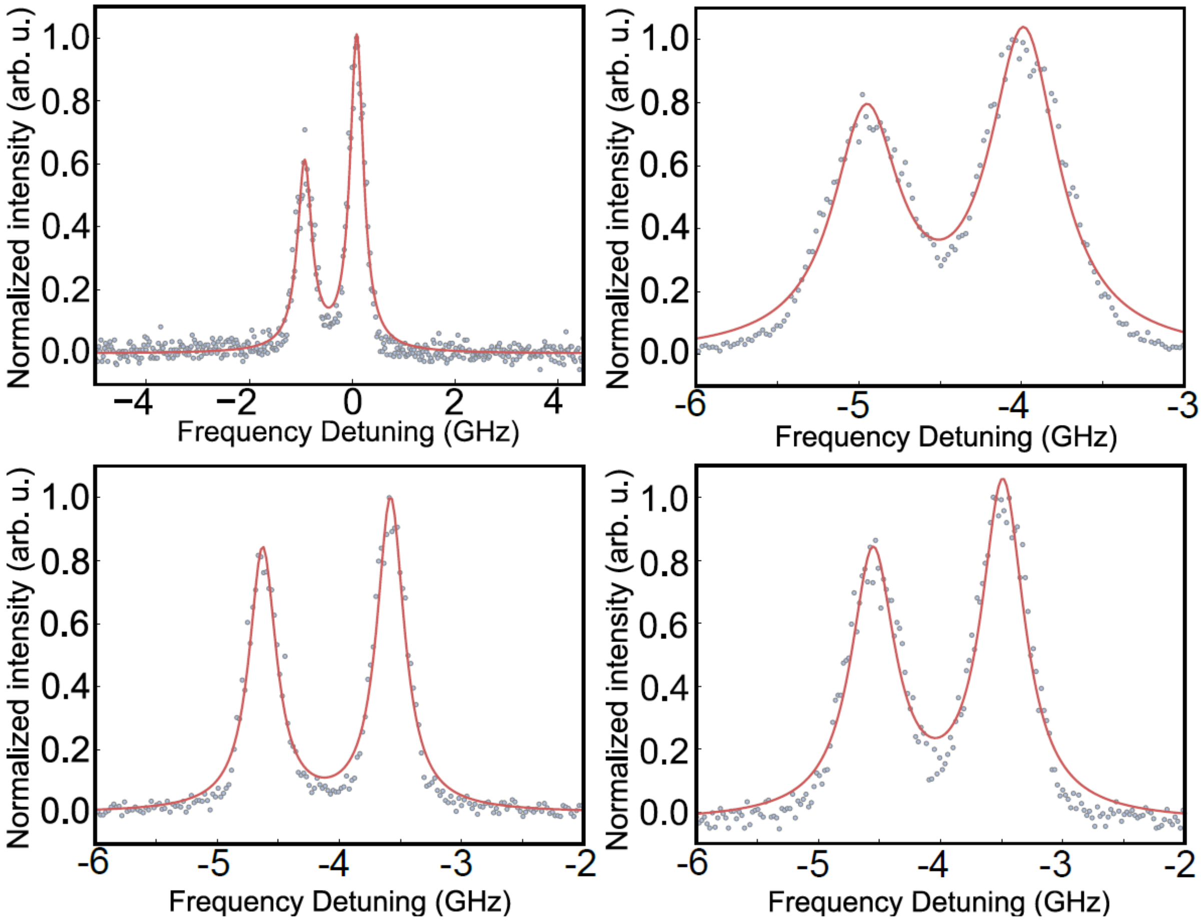

Supplementary Figure 2: A representing absorption spectrum of single V_{Si} centres in a high dose irradiated ($10^{13}\ cm^{-3}$) 4H-SiC sample.

Supplementary Note 5. Ab initio polarisation calculation for the negatively charged silicon vacancy centre in 4H-SiC.

In the main text, we report studies on resonant optical excitation spectra that show an outstanding spectral stability in contrast to the nitrogen-vacancy (NV) centre in diamond[6]. We attribute the small inhomogeneous distribution (see Figure 1(d) in the main text) to a low sensitivity of the defects to surrounding electric field fluctuations originating from other defects. Since this may be related to a small dipole moment of the V1 centre in 4H-SiC, we test this hypothesis by performing theoretical calculations as described in the following. We calculate the change in the polarisation for the excitation process between $^4A_2$ ground and $^4A_2$ excited states of the negatively charged V1 centre in 4H-SiC (silicon vacancy defect on a hexagonal lattice site). We compare these results with the ones obtained for the nitrogen-vacancy (NV) centre in diamond.

Computational details

We apply density functional theory (DFT) for electronic structure calculation and geometry relaxation using the plane-wave-based Vienna Ab initio Simulation Package (VASP)[7–10]. The core electrons are treated in the projector augmented-wave formalism[11]. For the 4H-SiC supercell, calculations are performed with 420 eV plane wave cut-off energy and with Γ centred $2 \times 2 \times 2$ k-point mesh to sample the Brillouin zone. For the diamond supercell, we use 420 eV plane wave cut-off energy and Γ-point to sample the Brillouin zone. We apply Perdew-Burke-Ernzerhof functional in these calculations[12]. The model for the silicon vacancy defect in bulk 4H-SiC is constructed using a 432-atom hexagonal supercell, whereas we use the 512-atom simple cubic supercell to model the NV centre in diamond. The excited state electronic structure and geometry is calculated by constraint occupation of states, or Delta Self-Consistent Field (ΔSCF) method[13].

We calculate the permanent polarisation in ground and excited states, and their difference, in order to infer the coupling to the optical transition. To this end, we use the VASP implementation of both Born effective charge calculation using density functional perturbation theory (DFPT)[14] and the Berry phase theory of polarization[15–17]. In

a DFT calculation, one can define the change in macroscopic electronic polarisation (P) as an adiabatic change in the Kohn-Sham potential (V_{KS})

$$\frac{\partial P}{\partial \lambda} = \frac{-\mathrm{i}fe\hbar}{\Omega m_{\mathrm{e}}} \sum_k \sum_{n=1}^{M} \sum_{m=M+1}^{\infty} \frac{\left\langle \psi_{kn}^{(\lambda)} \middle| \hat{\vec{p}} \middle| \psi_{km}^{(\lambda)} \right\rangle \left\langle \psi_{km}^{(\lambda)} \middle| \frac{\partial V_{\mathrm{KS}}}{\lambda} \middle| \psi_{kn}^{(\lambda)} \right\rangle}{\left(\varepsilon_{kn}^{(\lambda)} - \varepsilon_{km}^{(\lambda)} \right)^2} + c.c., \quad (4)$$

where f is the occupation number, e the elemental charge, m_{e} the electron mass, Ω the cell volume, M the number of occupied bands, $\vec{p}$ the momentum operator, λ is the adiabatic parameter, ε is the band energy. The first part of the equation corresponds to the electronic part of the permanent polarisation (p_{el}), whereas the second part corresponds to the contribution of ions (p_{ion}) to the permanent polarisation. In a periodic gauge, where the wavefunctions are cell-periodic and periodic in the reciprocal space, the permanent polarisation takes a form similar to the Berry phase expression

$$\Delta P = \frac{\mathrm{i}fe}{8\pi^3} \sum_{n=1}^{M} \int_{\mathrm{BZ}} dk \langle u_{kn} | \nabla_k | u_{kn} \rangle. \quad (5)$$

Using DFPT, $\nabla_k | u_{kn} \rangle$ can be calculated from the Sternheimer equations with similar self-consistent iterations as in DFT:

$$(H_k - \varepsilon_{kn} S_k) \nabla_k | u_{kn} \rangle = \frac{-\partial (H_k - \varepsilon_{kn} S_k)}{\partial k} | u_{kn} \rangle. \quad (6)$$

We determine the radiative transition rate between the ground and excited $^4A_2$ states by calculating the energy dependent dielectric function $\varepsilon_{\mathrm{r}}(E)$. The spontaneous transition rate is given by the Einstein coefficient

$$A = \frac{n\omega^3 |\mu|^2}{3\pi\varepsilon_0 \hbar c^3}, \quad (7)$$

where n is the refractive index, $\hbar\omega$ is the transition energy, μ is the optical transition dipole moment, ε_0 is the vacuum permittivity, and c is the speed of light. μ is proportional to the integrated imaginary dielectric function (I) of the given transition:

$$|\mu|^2 = \frac{\varepsilon_0 V}{\pi} \int \Im \varepsilon_{\mathrm{r}}(E) dE = \frac{\varepsilon_0 V I}{\pi}, \quad (8)$$

where V is the volume of the supercell.

Theoretical results

The results of the Berry phase evaluation for macroscopic dipole moment calculation are shown in Supplementary Tables 1 and 2 for the V1 centre in 4H-SiC and the NV centre in diamond, respectively. The change in the total dipole moment is about 20 times larger for NV centre in diamond with respect to that for V1 centre in 4H-SiC. This means that the V1 centre has intrinsically low coupling strength between optical transition and stray electric fields.

Supplementary Table 1: Macroscopic electric dipole moment of the hexagonal lattice site silicon vacancy defect (V1 centre) as calculated within the Berry phase approximation.

Transition	$\Delta p_{\mathrm{ion}}(e\mathrm{Å})$	$\Delta p_{\mathrm{el}}(e\mathrm{Å})$	$\Delta p_{\mathrm{tot}}(e\mathrm{Å})$
gr → ex (V1)	0	0.044	0.044

Supplementary Table 2: Macroscopic electric dipole moment of the NV centre in diamond as calculated within the Berry phase approximation.

Transition	$\Delta p_{\mathrm{ion}}(e\mathrm{Å})$	$\Delta p_{\mathrm{el}}(e\mathrm{Å})$	$\Delta p_{\mathrm{tot}}(e\mathrm{Å})$
gr → ex	0.061	0.842	0.903

Experimental results and discussion

Preliminary studies have been performed to constrain Δp_{tot} via Stark shift control of optical transition frequencies. To this end, we spanned two parallel copper wires over the sample in order to apply an electric field. The wires were separated by approximately 100 µm and voltages up to $\pm 200\,\mathrm{V}$ were applied. We note that by applying higher electric fields led to electrical breakdown in the cryostats low-vacuum atmosphere. Considering the relative permittivity of 4H-SiC $(\epsilon_{\mathrm{r}} \approx 10)$, this results in an estimated in-crystal field of about $\pm 200\,\frac{\mathrm{kV}}{\mathrm{m}}$. Two experiments were performed, one in which the electric field was applied along the crystal's c-axis, and a second one in which the field was orthogonal to the c-axis. We performed resonant excitation studies at zero magnetic field $(B_0 = 0\ \mathrm{G})$ as a function of the electric field strength, in analogy to the studies shown in Figure 1(c). A change in the width or separation of the A_1 and A_2 optical transitions would indicate electric field sensitivity through which Δp_{tot} can be inferred. We observed, however, neither effect within the investigated electric field range. In our current experimental setup, we can infer peak width and separation with a precision of approximately $\pm 12\ \mathrm{MHz}$. According to theory, an electric field perpendicular to c-axis has no coupling to V1 centre and the coupling is maximum with direction of the electric field parallel to c-axis. By assuming an homogeneous crystal field along the c-axis of $\pm 200\frac{\mathrm{kV}}{\mathrm{m}}$, we can constrain the Stark shift tuning coefficient to be $\approx 60\frac{\mathrm{MHz}}{\frac{\mathrm{MV}}{\mathrm{m}}}$. This is approximately two orders of magnitude smaller than reported for NV centres in diamond ($\approx 6.3\frac{\mathrm{GHz}}{\frac{\mathrm{MV}}{\mathrm{m}}}$)[18], such that we estimate $\Delta p_{\mathrm{tot}} < 0.009$. However, theory implies that the coupling coefficient is about an order magnitude smaller for V1 centre in SiC than that for NV centre in diamond. We show below that a compensating field can be developed when both electric field and illumination are applied that can explain the experimental data.

We note that there is a variation in the position of zero-phonon-line (ZPL) for various single V1 centres in the SiC sample of about few 100 MHz but the stability of the ZPL of each investigated single V1 centre is within 60 MHz after repetitive PLE measurements. This implies that charge redistribution occurs upon illumination for each single V1 centre even without applying external electric field. Understanding of these features

requires a close inspection of the SiC sample. First of all, the nitrogen donor concentration is very low ($\sim 3.5 \cdot 10^{13}\ \mathrm{cm}^{-3}$), thus the resistivity is very high. This means that the exchange of carriers is very limited, and the concept of Fermi-level does not apply to the entire SiC sample. Si-vacancies are created by 2 MeV irradiation of electrons. According to previous studies[19], the energy threshold of kicking off C and Si atoms in SiC lattice is about 120 keV and 250 keV, respectively. The application of 2 MeV energy should lead to a cascade process where the Si or C atoms kick out other C and Si atoms from the lattice. After annealing at 300 °C, the Frenkel-pairs disappear in the form of either recrystallization or antisite defects. The antisite defects are not activated by near infrared illumination. Besides, carbon interstitial clusters and vacancies may form. Interestingly, Si-vacancy has two faces in SiC crystal: the simple Si-vacancy which is basically a deep acceptor and the carbon antisite-vacancy pair (CAV) complex which has donor levels. Furthermore, the amphoteric carbon vacancies are left after the materials processing. Carbon vacancies are expected to appear in majority because they have the lowest formation energy among the considered defects[5] and threshold energy to form by electron irradiation[19]. The negative charge of the Si-vacancy should be donated by a nearby defect. The average distance between nitrogen donors is about 306 nm in the SiC sample, thus only few Si-vacancies will reside near (~50 nm) the nitrogen donor. Among the considered vacancy-like defects, the CAV defect has the shallowest donor level[5], which can be activated by near infrared illumination. The simplest model is that a positively charged defect resides near the negatively charged Si-vacancy, the V1 centre. According to our calculations, the positively charged donor defect (N-donor or CAV) should sit around ~40 nm around the V1 centre at different lattice sites going from the symmetry axis of the defect toward the basal plane, in order to experience few 100 MHz variation in the ZPL of various V1 centres. The small spectral diffusion may be understood by assuming that another donor defect lies near the V1 centre with about the same distance but another location where the illumination will activate that donor, and the resulting electron, free carrier, will be captured by the previously positively charged donor defect. According to a previous study[20], low energy Si atoms will produce vacancy defects in about 10 nm region; thus Si atoms that are created by 2 MeV electron irradiation have much higher kinetic energy and should produce vacancies, antisites and interstitials at larger distances, around 40 nm and larger distances. By applying an external electric field that is parallel

to the symmetry axis of the V1 centre, illumination will again ionize a donor defect but the electric field will drag the electron in the opposite direction of the electric field. One of the carbon vacancies around V1 centre will capture this electron, and the positive donor and negative carbon vacancy will form an electric field that mostly screens the external electric field. We find that if these defects are both 40 nm apart from the V1 centre along the symmetry axis then they shield the external electric field to about 10% of its magnitude. As a consequence, the resulting Stark-shift agrees with the experimental data ($\Delta p_{\mathrm{tot}} < 0.09$). Although, this estimation is crude as the statistics about the point defects around V1 centre is not known but our scenario still explains all the experimental findings. We think that both the small coupling constant of the V1 centre and the shielding effects created by the donor and acceptor point defects around the V1 centre are responsible for the spectral stability of the V1 centre.

Supplementary Note 6. Electronic fine structure and spin polarization of the V1 defect.

A simplified electronic fine structure model of the V1 defect is shown in Supplementary Figure 3. The spin $m_S = \pm 1/2$ and $m_S = \pm 3/2$ states of the first excited state (es) are labeled as es_1 and es_2, respectively. Similarly, the spin states $m_S = \pm 1/2$ and $m_S = \pm 3/2$ belonging to the ground state (gs) are denoted by gs_1 and gs_2. The optically active transitions $es_1 \to gs_1$ (red transition) and $es_2 \to gs_2$ (blue transition) have equal radiative decay rates as the states involved in these transitions have the same

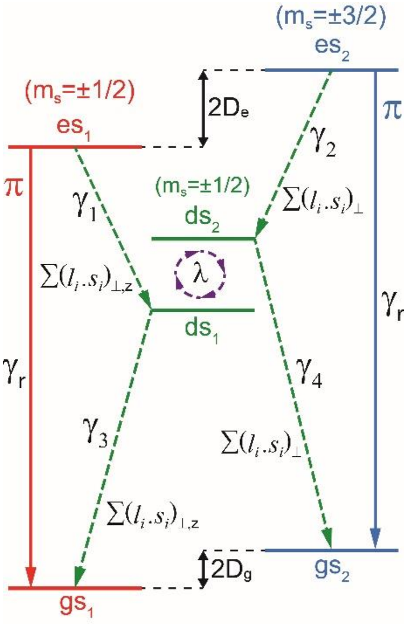

Supplementary Figure 3: Electronic fine structure of the V1 defect in 4H-SiC.

molecular orbital configurations resulting with equal transition dipole moments. Moreover, the spin polarisation of the V1 defect is governed by the phonon-assisted non-radiative intersystem crossing (ISC) from and to the metastable doublet states, ds_1 and ds_2, via direct spin-orbit coupling (DSO).

The four non-radiative ISC decay rates shown in Supplementary Figure 3 determine the unique features in the PLE spectra of this defect. The non-radiative decay rates in the entry channel that are associated with the decay from es_1 to ds_1 and from es_2 to ds_2 are labeled as γ_1 and γ_2, respectively, and they determine the amplitudes of the A_1 and A_2 peaks shown in Figure1(c). Similarly, the decay rates originating from ds_1

back to gs1 and from ds2 back to gs2 are labeled as γ_3 and γ_4, respectively, and they are responsible for the shelving lifetime of this defect.

We obtain the relationships between γ_1 and γ_2, as well as γ_3 and γ_4, by evaluating the corresponding DSO matrix elements, $\gamma_i = \frac{2\pi}{\hbar}\left|\langle\psi_i^{\mathrm{gs}}|\Sigma_j\, l_j.s_j|\psi_i^{\mathrm{ds}}\rangle\right|^2$, in the symmetry adapted $\psi_i^{\mathrm{ds/gs}}$ wave-functions basis of $j = 5$ active electrons[27]. We find that 2 metastable doublet states (with symmetries A_1 and 2E) have non-zero DSO matrix elements that can participate in the ISC. The E-symmetry ds (e^3) can couple to both es and gs by the orthogonal component of the DSO $l_{\perp}.s_{\perp}$ (with respect to the c-axis). The remaining A1 ds (ve^2) is strongly hybridized with the E ds (e^3) by the $l_{\parallel}.s_{\parallel}$ component of the DSO. As a result, the overall ISC can be simplified into two doubly degenerate metastable states, labeled as ds1 and ds2, which can only couple to the spin $\pm 1/2$ or the $\pm 3/2$ states of the es and gs, respectively.

The PLE spectra of the V1 defect can be accurately reproduced by using the theoretical fine structure of Supplementary Figure 3. The PLE signal of the V1 defect corresponds to the sum of the steady state es1 and es2 excited state populations of es1 and es2, that are calculated using the following master equation,

$$\frac{d\rho}{dt} = -\frac{i}{\hbar}[H_0,\rho] + \gamma_r \sum_{\mathrm{i}=0}^{2} L(A_{\mathrm{r}}^{i}) + \sum_{\mathrm{i}=0}^{4} \gamma_i L(A_{\mathrm{ds}}^{i}) + \gamma_{\mathrm{R}} \sum_{\mathrm{i}=0}^{2} L(A_{\mathrm{R}}) + \gamma_{\mathrm{s}} L(A_{\mathrm{s}}). \qquad (9)$$

The decay and decoherence processes of the defect are represented by the Lindblad super-operators, *i.e.* $L(O) = O\rho O^{+} - \{O^{+}O,\rho\}/2$ for any operator *O*. The electronic fine structure Hamiltonian is given by,

$$H_0 = \frac{(D_{\mathrm{gs}}-D_{\mathrm{es}}+\delta_L)}{2}\left(|gs_1\rangle\langle gs_1| - |es_1\rangle\langle es_1|\right) - \frac{(D_{\mathrm{gs}}-D_{\mathrm{es}}-\delta_{\mathrm{L}})}{2}\left(|gs_2\rangle\langle gs_2| - |es_2\rangle\langle es_2|\right) + \lambda\left(|ds_1\rangle\langle ds_2| + |ds_2\rangle\langle ds_1|\right) + \Omega_{\mathrm{L}}\left(|gs_1\rangle\langle es_1| + |gs_2\rangle\langle es_2| + c.c.\right) \qquad (10)$$

in the rotating frame of the excitation laser with Rabi frequency Ω_{L} and detuning $\delta_{\mathrm{L}} = \omega_{\mathrm{L}} - \omega_{\mathrm{ZPL}}$ from the zero phonon line (ZPL). The excited and ground state's ZFS are given by $2D_{\mathrm{es}}$ (975 MHz) and $2D_{\mathrm{gs}}$ (9 MHz), respectively. The radiative decays, $A_{\mathrm{r}}^{i} = |gs_i\rangle\langle es_i|$, have same radiative decay rate γ_{r}. The ISC from es to the metastable

ds are given by $A_{\mathrm{ds}}^1 = |ds_1\rangle\langle es_1|$ and $A_{\mathrm{ds}}^2 = |ds_2\rangle\langle es_2|$ with rates γ_1 and γ_2, whereas the ISC from ds back to gs are denoted by $A_{\mathrm{ds}}^3 = |gs_1\rangle\langle ds_1|$ and $A_{\mathrm{ds}}^4 = |gs_2\rangle\langle ds_2|$ with rates γ_3 and γ_4, respectively. The intrinsic spin relaxation in the ground state and dephasing among ds are considered by $A_{\mathrm{R}} = |gs_1\rangle\langle gs_2|+|gs_2\rangle\langle gs_1|$ with rate γ_{R} and $A_{\mathrm{s}} = |ds_1\rangle\langle ds_1| - |ds_2\rangle\langle ds_2|$ with rate γ_{s}. Spin-mixing among ds via fast relaxation processes is also included in H_0 by the λ term.

Resonant optical excitation along the A_2 transition ($\mathrm{gs}_2 \rightarrow \mathrm{es}_2$) will lead to an optical pumping into the gs_1. Therefore, to observe a measurable signal during PLE, the ground state spin must be able to relax. Alternatively, one can use MW pulses on the gs spin states for coherent control as well as to overcome this optical pumping. During a continuous broadband MW pulse where all three gs spin transitions shown in Figure 2(a) are allowed to relax equally, the PLE amplitude mismatch between A_1 and A_2 is directly determined by the ISC rates of γ_1 and γ_2. We find that the faster γ_1 rate causes more population to be removed non-radiatively from $\left|\pm\frac{1}{2}\right\rangle_{\mathrm{es}}$ per optical cycle compared to $\left|\pm\frac{3}{2}\right\rangle_{\mathrm{es}}$, therefore leading to a smaller amplitude for A_1 than A_2. By comparing the theoretically calculated PLE signal shown in Supplementary Figure 4 to our experimental results, we deduce that $\gamma_1 \cong 3\gamma_2$.

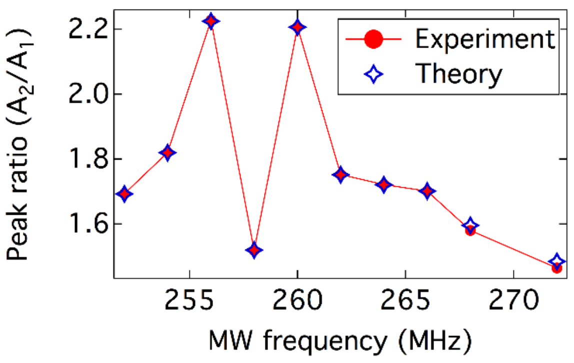

Supplementary Figure 4: Calculated and measured A_2/A_1 ratios for various broadband MW frequencies.

The es ZFS sign, which is determined as in the above section (S3), can also be confirmed by comparing this model to a series of MW schemes in the presence of a magnetic field $B_0 = 96\ \mathrm{G}$ (*i.e.* $g\mu_\mathrm{B} B_0 S_\mathrm{z} \gg 2D_\mathrm{gs}$). In these schemes, a continuous broadband MW is scanned from 252MHz to 272MHz and at each frequency the MW centered the A_2/A_1 peak ratio is determined from the corresponding PLE spectra. At 258 MHz, all three spin transitions of the ground state (see Figure 2(a)) are allowed to depopulate and the A_2/A_1 ratio is solely determined by the γ_1 and γ_2 ISC rates. On the other hand, MWs centered at 256 MHz and 260 MHz cannot effectively repopulate the $\left|+\frac{1}{2}\right\rangle_\mathrm{gs}$ and $\left|-\frac{1}{2}\right\rangle_\mathrm{gs}$, respectively. This results with increased optical pumping into $\left|+\frac{3}{2}\right\rangle_\mathrm{gs}$ or $\left|-\frac{3}{2}\right\rangle_\mathrm{gs}$ state, and causes a significant reduction in the $\left|\pm\frac{1}{2}\right\rangle_\mathrm{es} \to \left|\pm\frac{1}{2}\right\rangle_\mathrm{gs}$ peak amplitude. Therefore, the observed increase in the A_2/A_1 ratio for MW frequencies 256 MHz and 260 MHz further supports the positive sign assignment of the es ZFS since it identifies the A_1 peak as the $\left|\pm\frac{1}{2}\right\rangle_\mathrm{es} \to \left|\pm\frac{1}{2}\right\rangle_\mathrm{gs}$ transition.

Supplementary Note 7. Decoherence sources limiting spin coherence times.

In the main text, we report a spin decoherence time of $T_2 = 0.85 \pm 0.12\ \mathrm{ms}$, measured by Hahn echo. Although this result is better than the previously reported values[4,21–23], one may anticipate reduced decoherence rates owing to the use of an isotopically purified nuclear spin free 4H-SiC sample, in analogy to previous experiments with isotopically purified diamond and silicon[24,25].

As we used a rather low dose of electron beam irradiation to create defect centres, the concentration of paramagnetic defects is small $(\sim 10^{13}\ \mathrm{cm}^{-3})$ as explained in Methods section, which cannot explain the observed T_2 times. Shallow nitrogen donors are also discarded as a major decoherence source as their concentration is also too low to be significant $(\sim 3.5 \cdot 10^{13}\ \mathrm{cm}^{-3})$ since the equivalent spin dipole-dipole interaction in electronic spin bath requires higher total impurity concentration ($\sim 6 \cdot 10^{14}\ \mathrm{cm}^{-3}$)[21].

We therefore attribute the main decoherence source to be undesired defects near surface paramagnetic defects created by cutting with a typical concentration in the low 10^{13} cm^{-2} range in the region about ~1 µm from the surface[26]. Before irradiation, samples were annealed to 1130 °C in N_2 gas flow to reduce the concentration of paramagnetic surface defects to below detection of Electron Paramagnetic Resonance (EPR) (below 10^{12} cm^{-2}). Since the N_2 annealing is known to reduce only 10% of the surface defects and the EPR experimental conditions are not optimized for the detection of the surface defects, it is safe to assume that the surface defect concentration within ~1 μm range from the surface has an upper limit of 10^{16} cm^{-3}. As the optical transition dipole of the investigated defect centres is parallel to the crystal's c-axis, we had to flip the sample by 90°. This comes with the trade-off that all observed defects are located close to the cutting surface. The cutting surfaces are also expected to have structural defects induced by micro cracks caused by cutting. In the future, this issue can be addressed by improved sample processing[26] or by growing SiC layers on a-plane substrate so that solid immersion lens can be fabricated on as-grown surfaces, which contain no such defects.

Supplementary Note 8. Additional data for orientation and polarisation of the optical transitions.

Previous studies have already shown that the silicon vacancy centre at a hexagonal lattice site (V1 centre) is most effectively excited using a linearly polarised off-resonant laser whose polarisation is parallel to the crystal's c-axis[3,4].

Here, we investigate the behaviour of the individual optical transitions A_1 and A_2 under resonant excitation. As we have shown in the main text, when applying a magnetic field, no additional optical transitions are observed, supporting the absence of circularly polarised optical transitions.

In the following, we show that both transition dipoles are indeed linearly polarised and parallel to each other. To this end, we applied broadband microwaves in order to continuously mix the ground state populations, and performed resonant optical excitation along either A_1 or A_2 at an intensity of about $1\ \mathrm{W\,cm^{-2}}$. The polarisation of the excitation laser was adjusted by a half-wave plate (HWP), the efficiency of the excitation was inferred from the detected fluorescence intensity in the phonon sideband. Supplementary Figure 5 shows the experimental results. In both cases, tuning the HWP leads to near-perfect sinusoidal oscillations, demonstrating that linear dipole transitions are excited, which are parallel to each other.

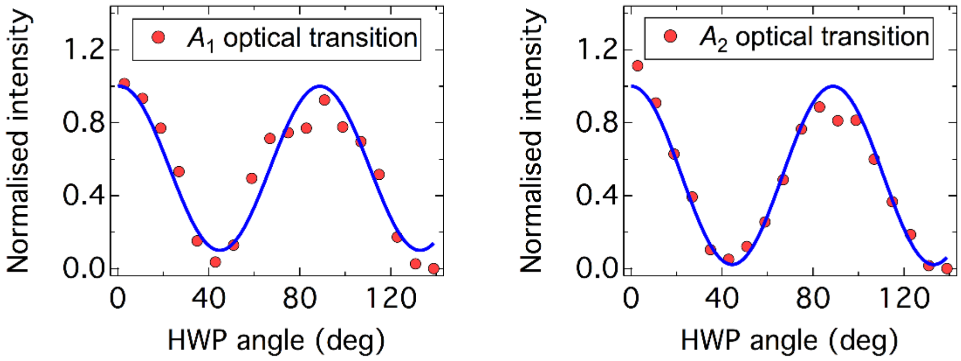

Supplementary Figure 5: Measuring the polarisation of the optical dipole transitions A_1 (left side) and A_2 (right side). Dots are data, and blue lines are sinusoidal fits. The high contrast in the observed oscillations shows that both dipoles are linear and parallel to each other.

Supplementary References

1. Brown, R. H. & Twiss, R. Q. LXXIV. A new type of interferometer for use in radio astronomy. *Philos. Mag.* **45,** 663–682 (1954).
2. Kitson, S. C., Jonsson, P., Rarity, J. G. & Tapster, P. R. Intensity fluctuation spectroscopy of small numbers of dye molecules in a microcavity. *Phys. Rev. A* **58,** 620–627 (1998).
3. Janzén, E. *et al.* The silicon vacancy in SiC. *Phys. B Condens. Matter* **404,** 4354–4358 (2009).
4. Nagy, R. *et al.* Quantum Properties of Dichroic Silicon Vacancies in Silicon Carbide. *Phys. Rev. Appl.* **9,** 34022 (2018).
5. Ivády, V. *et al.* Identification of Si-vacancy related room temperature qubits in 4H silicon carbide. *Phys. Rev. B* **96,** 161114(R) (2017).
6. Siyushev, P. *et al.* Optically Controlled Switching of the Charge State of a Single Nitrogen-Vacancy Center in Diamond at Cryogenic Temperatures. *Phys. Rev. Lett.* **110,** 167402 (2013).
7. Kresse, G. & Furthmüller, J. Efficient iterative schemes for ab initio total-energy calculations using a plane-wave basis set. *Phys. Rev. B* **54,** 11169–11186 (1996).
8. Kresse, G. & Hafner, J. Ab initio molecular dynamics for liquid metals. *Phys. Rev. B* **47,** 558–561 (1993).
9. Kresse, G. & Furthmüller, J. Efficiency of ab-initio total energy calculations for metals and semiconductors using a plane-wave basis set. *Comput. Mater. Sci.* **6,** 15–50 (1996).
10. Paier, J. *et al.* Screened hybrid density functionals applied to solids. *J. Chem. Phys.* **124,** 154709 (2006).
11. Blöchl, P. E. Projector augmented-wave method. *Phys. Rev. B* **50,** 17953–17979 (1994).
12. Perdew, J. P., Burke, K. & Ernzerhof, M. Generalized Gradient Approximation Made Simple. *Phys. Rev. Lett.* **77,** 3865–3868 (1996).

13. Gali, A., Janzén, E., Deák, P., Kresse, G. & Kaxiras, E. Theory of Spin-Conserving Excitation of the $N\ensuremath{-}{V}^{\ensuremath{-}}$ Center in Diamond. *Phys. Rev. Lett.* **103,** 186404 (2009).

14. Gajdoš, M., Hummer, K., Kresse, G., Furthmüller, J. & Bechstedt, F. Linear optical properties in the projector-augmented wave methodology. *Phys. Rev. B* **73,** 45112 (2006).

15. King-Smith, R. D. & Vanderbilt, D. Theory of polarization of crystalline solids. *Phys. Rev. B* **47,** 1651–1654 (1993).

16. Vanderbilt, D. & King-Smith, R. D. Electric polarization as a bulk quantity and its relation to surface charge. *Phys. Rev. B* **48,** 4442–4455 (1993).

17. Resta, R. Macroscopic polarization in crystalline dielectrics: the geometric phase approach. *Rev. Mod. Phys.* **66,** 899–915 (1994).

18. Tamarat, P. *et al.* Stark Shift Control of Single Optical Centers in Diamond. *Phys. Rev. Lett.* **97,** 83002 (2006).

19. Steeds, J. W. *et al.* Differentiation between C and Si Related Damage Centres in 4H- and 6H-SiC by the Use of 90-300 kV Electron Irradiation Followed by Low Temperature Photoluminescence Microscopy. *Mater. Sci. Forum* **353–356,** 381–384 (2001).

20. Wallace, J. B., Bayu Aji, L. B., Shao, L. & Kucheyev, S. O. Time constant of defect relaxation in ion-irradiated 3 C -SiC. *Appl. Phys. Lett.* **106,** (2015).

21. Widmann, M. *et al.* Coherent control of single spins in silicon carbide at room temperature. *Nat Mater* **14,** 164–168 (2015).

22. Carter, S. G., Soykal, Ö. O., Dev, P., Economou, S. E. & Glaser, E. R. Spin coherence and echo modulation of the silicon vacancy in 4H-SiC at room temperature. *Phys. Rev. B* **92,** 161202(R) (2015).

23. Simin, D. *et al.* Locking of electron spin coherence above 20 ms in natural silicon carbide. *Phys. Rev. B* **95,** 161201 (2017).

24. Balasubramanian, G. *et al.* Ultralong spin coherence time in isotopically engineered diamond. *Nat Mater* **8,** 383–387 (2009).

25. Tyryshkin, A. M. *et al.* Electron spin coherence exceeding seconds in high-purity silicon. *Nat Mater* **11,** 143–147 (2012).

26. Zvanut, M. E., Thomas, S. & Dashdorj, J. Intrinsic Surface Defects on 4H SiC Substrates. *MRS Proc.* **1246,** 1246-B03-03 (2010).

27. Soykal, Ö. O., Dev, P. & Economou, S. E. Silicon vacancy center in 4H-SiC: Electronic structure and spin-photon interfaces. *Phys. Rev. B* **93,** 081207 (2016).
